# 79512 The Great Plains IDeA CTR Research Scholar Program

**DOI:** 10.1017/cts.2021.772

**Published:** 2021-03-30

**Authors:** Ted Mikuls, Heather Braddock, Lani Zimmerman

**Affiliations:** University of Nebraska Medical Center

## Abstract

ABSTRACT IMPACT: To date, our Scholars have been highly productive in the conduct of impactful research and have contributed to the literature through dissemination of their findings. OBJECTIVES/GOALS: The primary purpose of the Great Plains IDeA Research Scholars Program (RSP) is to support the development and retention of early-career faculty preparing to compete for external funding as clinical-translational research (CTR) investigators. We developed processes for RSP applications, prioritization, and selection criteria. METHODS/STUDY POPULATION: In year 1, we admitted 4 Scholars and have since added 5 additional Scholars. Scholars are retained in the program until they receive R- or K-level funding or if progress is deemed to be substandard on two consecutive 6-month reviews (no awards revoked to date). Each scholar was assigned a mentor(s) or mentoring team. Each participant developed 1-year goals and a 4-5-year plan that included a refined proposal to collect preliminary data, a timetable for grant submissions (with a focus on R01 applications or equivalent), and personal goals to enhance chances of success. Scholars composed an Individual Development Plan (IDP) with mentor(s) feedback to identify the skills needed to achieve goals. Each Scholar completed pilot work to generate the requisite preliminary data for an extramural grant application. RESULTS/ANTICIPATED RESULTS: Over four years, we have had 9 scholars from 4 sites with 51 total applications. All scholars completed required grant writing seminar courses, an 8-hour Responsible Conduct of Research course, and were given access to seminars and workshops sponsored by the Great Plains IDeA CTR. Scholars received 0.5 FTE in research support and $50,000 annual funding to support their research and/or career development activities. Seven of 9 scholars have completed the program to date, collectively receiving an R01 (1), a U01 (1), a K23 (1), a VA Career Development Award (1), or COBRE pilot projects leadership roles (3). All remain active faculty at their institution. Two remaining scholars are working towards independent funding: both have submitted extramural grant applications with anticipated funding this year. DISCUSSION/SIGNIFICANCE OF FINDINGS: To date, our Scholars have been highly productive in the conduct of impactful research. They have submitted 36 grants; culminating in 27 funded projects and >$7.5 million in total funding for 7 graduates. They have disseminated their findings through 121 publications and 48 invited regional/national presentations.

